# Mode of Delivery Among Women with a History of Prior Cesarean Birth at Mizan-Tepi University Teaching Hospital

**DOI:** 10.26502/fjwhd.2644-28840055

**Published:** 2021-01-08

**Authors:** Margo S Harrison, Tewodros Liyew, Ephrem Kirub, Biruk Teshome, Andrea Jimenez-Zambrano, Margaret Muldrow, Teklemariam Yarinbab

**Affiliations:** 1 University of Colorado School of Medicine, Colorado, USA; 2 Mizan-Tepi University Teaching Hospital, Aman, Bench Maji Zone, Ethiopia; 3 Village Health Partnership, Colorado, USA

**Keywords:** History of Cesarean Birth, Ethiopia

## Abstract

**Objectives:**

The objective of this study was to observe mode of delivery among women with a history of prior cesarean birth.

**Methods:**

After collecting data on a convenience sample of 1,000 women giving birth at 28 weeks gestation or greater at Mizan-Tepi University Teaching Hospital, we reduced the sample to only include women with a history of prior cesarean birth. We wanted to observe mode of delivery among this cohort and determine if any characteristics were associated with elective repeat cesarean birth, as compared to vaginal birth after cesarean.

**Results:**

Of 1,000 women in our convenience sample, data on history of prior cesarean birth was missing on 2 women (0.2%). Of the remaining women, 49 (4.9%) reported a history of prior cesarean; 44 (89.8%) reported one prior cesarean and 5 (10.2%) women had two prior cesarean births. Repeat cesarean birth occurred in 65.1% (n = 29/44) of women with one prior cesarean and in 80.0% (n = 4/5) of women with two prior surgeries. Among the total cohort of women with a history of prior cesarean birth, of those who experienced repeat cesarean birth (n = 33), 27.3% (n = 9) occurred pre-labor, 69.7% (n = 23) occurred intrapartum after the onset of spontaneous labor, and 3.0% (n = 1) occurred intrapartum during the course of an induced or augmented labor. Labor onset and cervical exam on admission were statistically significantly different in bivariate comparisons of women who successfully achieved vaginal birth after cesarean as compared to those who gave birth by repeat cesarean birth, and postpartum maternal antibiotics were more common after repeat cesarean birth, p < 0.05. In a multivariable model of factors associated with successful vaginal birth after cesarean, the likelihood of successful vaginal birth was increased 15% for each increasing centimeter of dilation on a woman’s admission cervical exam (RR 1.15, p= 0.004).

**Conclusion:**

Almost one-third of women in our observational cohort attempted trial of labor after cesarean; those that were successful were more likely to have been more cervically dilated on their admission exam. No sociodemographic or obstetrical characteristics were more likely among women who underwent pre-labor repeat cesarean birth as compared to intrapartum cesarean birth.

## 1. Introduction

Cesarean birth rates are increasing, globally [1], As cesarean births rates increase, so does the global prevalence of women with a history of prior cesarean birth. There is equipoise between whether trial of labor after cesarean versus elective repeat cesarean birth should be recommended among this population as each mode of delivery is associated with a balance of risks and benefits for the mother, as well as for the fetus [2]. It is increasingly common to focus on informed decision making with women regarding their delivery options [2], Decision aids and predictive models have been devised to assist with this process [3–5]. Little data on these practices in low- and middle-income countries are available. In order to detennine the need for assistance with decision-making in low-resource settings, it is imperative to first understand what mode of delivery women with a history of prior cesarean birth are experiencing. We know from prior research that rates of vaginal birth after cesarean in sub-Saharan Africa are around 80.0%; thus, we hypothesized that among our convenience sample of women giving birth at a teaching and referral facility in the Southern Nations, Nationalities, and People’s Region of Ethiopia, we would expect to observe similar rates [6]. We wanted to determine if any characteristics were associated with mode of delivery (repeat cesarean birth versus vaginal birth after cesarean), and if any outcomes were more likely after one mode compared to the other.

## 2. Methodology

We conducted a hospital-based, prospective, cross-sectional quality improvement analysis at Mizan-Tepi University Teaching Hospital (MTUTH), in the Southern Nations, Nationalities, and People’s Region of Ethiopia. We observed a convenience sample of 1,000 women who gave birth (after 28 completed weeks of gestation) on labor and delivery at MTUTH between May 6 and October 21, 2019. Through a combination of chart review and structured interview at admission, delivery, and discharge, three physicians collected de-identified data, which was entered into REDCap [7]. Bivariate comparisons of sociodemographic, obstetric, birth, and pregnancy outcomes of women experiencing vaginal versus cesarean birth were performed using STATA software version 15.2 (StataCorp LP, College Station, TX, USA). Fisher’s exact, Chi-squared, and Kruskal-Wallis tests were performed depending on the variables. All covariates significant to p < 0.05 were included in a multivariable Poisson model with robust error variance (because vaginal birth after cesarean birth was prevalent) to determine which covariates were independently associated with cesarean birth. Despite the quality improvement nature of the work and the fact that only de-identified data was collected, oral consent was obtained from each woman before any of her data was recorded. This quality improvement survey was given an exempt from human subjects’ research approval (COMIRB # 18-2738) by the University of Colorado and approval.

## 3. Results

**Figure 1:**
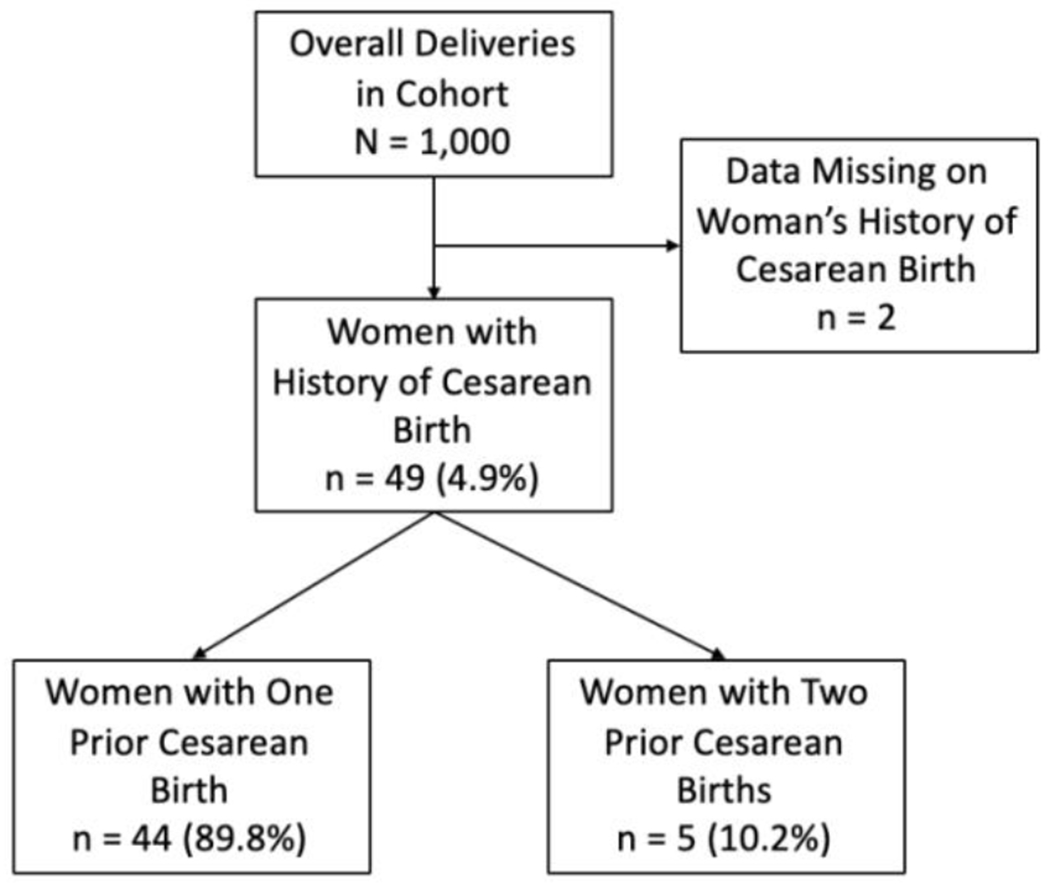
Population of women with history of prior cesarean birth at Mizan-Tepi University Teaching Hospital.

Figure 1 illustrates our study cohort. Of 1,000 women in our convenience sample, data on history of prior cesarean birth was missing on 2 women (0.2%). Of the remaining women, 49 (4.9%) reported a history of prior cesarean; 44 (89.8%) reported one prior cesarean and 5 (10.2%) women had two prior cesarean births. Table 1 shows mode of delivery (spontaneous vaginal birth, forceps-assisted or vacuum-assisted vaginal birth, and repeat cesarean birth) of the cohort of women with a history of prior cesarean birth. Repeat cesarean birth occurred in 65.1% (n = 29/44) of women with one prior cesarean and in 80.0% (n = 4/5) of women with two prior surgeries. Two of the women (4.6%) who achieved successful vaginal birth after a history of one prior cesarean did so with vacuum assistance, while there were no assisted vaginal births among women with a history of two prior cesareans. Table 2 illustrates when in the labor course birth occurred for women who delivered by repeat cesarean. Among the total cohort of women with a history of prior cesarean birth, of those who experienced repeat cesarean birth (n = 33), 27.3% (n = 9) occurred pre-labor, 69.7% (n = 23) occurred intrapartum after the onset of spontaneous labor, and 3.0% (n = 1) occurred intrapartum during the course of an induced or augmented labor. The table also shows the reported general indications for cesareans at the study site, which by order of prevalence were maternal (66.7%), fetal (24.2%), maternal and fetal (6.1%), and failed trial of labor (3.0%).

Table 3 compares women who experienced vaginal birth after cesarean to those who underwent repeat cesarean birth. Overall the women had a median age of 25 years (interquartile range [IQR] 22,29), 44.9% of them had a primary school education, 42.9% were Protestant, 100.0% were not single, 55.1% lived in an urban area, and the women experienced a median number of 4 prenatal visits (IQR 4,5). Almost two-thirds of the population was nulliparous, the median interpregnancy interval was 48 months (IQR 1.5,3], most were in labor less than twenty-four hours (78.5%), and antepartum hemorrhage and blood pressure elevations were prevalent in 5.4% and 6.1% of the cohort, respectively. The median gestational length was 39 weeks (IQR 38,42), only 2.0% of neonates weighed less than 2500 grams, and the sex imbalance was quite pronounced—males accounted for two-thirds of the population.

In bivariate comparisons, shown in columns 2 – 4 of Table 3, only onset of labor and cervical dilation on admission to the facility were found to be statistically significantly different between women experiencing repeat cesarean birth as compared to those experiencing vaginal birth after cesarean. All women (100.0%) who achieved vaginal birth after cesarean went into labor spontaneously, compared to 69.7% of those who underwent repeat cesarean birth. Women who delivered vaginally were admitted with a higher cervical dilation on admission (3 versus 2 centimeters) compared to those who were delivered by repeat cesarean. It was also notable that maternal postpartum antibiotic administration was higher in women who experienced repeat cesarean. The final rows of Table 3 shown multivariable modeling of characteristics associated with successful vaginal birth after cesarean. The only characteristic that was associated with a higher likelihood of vaginal birth was greater dilation on admission; each centimeter of increased dilation was associated with a 15% increase risk (chance) of vaginal birth (RR 1.15, p = 0.004).

## 4. Discussion

Our analysis showed that almost one-third of women with a history of prior cesarean birth are attempting a trial of labor after cesarean at MTUTH, including women requiring induction or augmentation. We have conducted a number of other analyses with this dataset on characteristics associated with cesarean birth among subgroups of women and have found that generally, there are clear obstetric complications associated with cesarean birth, such as prolonged labor. In this analysis, however, the characteristic associated with cesarean birth was how dilated (Bishop’s score) the woman was on admission—the more dilated she was, the lower her risk of cesarean birth. Reported indications were maternal, fetal, or a combination of the two, with one woman identified as undergoing repeat cesarean birth for failed trial of labor.

According to our analysis, there was no difference in the prevalence of intrapartum or postpartum complications between the study groups. We did test a number of antepartum, intrapartum, and postpartum maternal and neonatal complications, but there was no difference in outcomes between the groups (data not shown). Prior research has found that pre-labor elective research cesarean birth is associated with reduced postpartum hemorrhage and improved neonatal outcomes as compared to vaginal birth after cesarean [8]. Though the data are not shown, we did compare all pre-labor to intrapartum cesarean births (leaving out vaginal births after cesarean) and found no bivariate differences in antepartum, intrapartum, or postpartum characteristics or outcomes, either (data not shown). This suggests, along with the fact that 100.0% of women achieving vaginal birth after cesarean were in labor spontaneously, that women and/or providers may be selecting women for mode of delivery, appropriately. Those that are admitted in labor with a favorable exam may be allowed to labor, as compared to those who are not dilated and showing no signs of labor.

When we discussed these findings with coauthors and colleagues at MTUTH, the way these women are currently managed is that providers currently screen for any contraindications to vaginal birth after cesarean using a protocol based on national recommendations. If, according to the protocol, the woman qualifies for a safe trial of labor, she will then be offered the option of cesarean birth versus trial of labor after cesarean. She is usually counseled by a physician during her antenatal care in the outpatient setting and given time to consider the choice. No specific protocol is currently used for counseling. If she chooses to labor, she is monitored with electronic fetal monitoring or managed one-on-one by a midwife on service. MTUTH also has a blood bank on site. The current practices at MTUTH appear to be resulting in the safe management of trial of labor after cesarean. An area of research might be into the standardization of the vaginal birth after cesarean counseling women are receiving or use of a decision aid to ensure that all women are receiving similar information under similar circumstances. The one-to-one attention during trial of labor would also be an area of potential research. This analysis is limited by the lack of contextual data surrounding counseling of women and the method women use to decide on mode of delivery after a prior cesarean birth. Further qualitative research on this topic would add richness to the data and assist with disseminating the success that MTUTH has achieved regarding safe trial of labor after cesarean and vaginal birth after cesarean. Prospective research on the process used by MTUTH regarding mode of delivery decision-making and intrapartum management would enable further research on best methods to implement and disseminate these evidence-based practices, elsewhere.

## Figures and Tables

**Table 1:** Mode of delivery among women with a history of prior cesarean birth, by number of prior cesareans.

Method of Delivery	Women with One Prior Cesarean (n = 44, 89.8%)	Women with Two Prior Cesareans (n = 5, 10.2%)
Spontaneous Vaginal Birth	13 (29.5%)	1 (20.0%)
Forceps-Assisted Vaginal Birth	0 (0.0%)	0 (0.0%)
Vacuum-Assisted Vaginal Birth	2 (4.6%)	0 (0.0%)
Repeat Cesarean Birth	29 (65.1%)	4 (80.0%)

**Table 2:** Onset of labor and indication for cesarean birth among women delivered by repeat cesarean birth.

Onset of Labor	Women Delivered by Repeat Cesarean Birth (n = 33, 67.3%)
Pre-Labor	9 (27.3%)
Intrapartum, after Spontaneous Labor Onset	23 (69.7%)
Intrapartum, after Induction/Augmentation of Labor	1 (3.0%)
	**Indication for Cesarean for Women Delivered by Repeat Cesarean Birth (n = 33)**
Maternal Indication, Only	22 (66.7%)
Maternal & Fetal Indication	2 (6.1%)
Fetal Indication, Only	8 (24.2%)
Failed Labor	1 (3.0%)

**Table 3:** Bivariate comparisons of patient characteristics and outcomes between women undergoing repeat cesarean birth as compared to vaginal birth after cesarean, and multivariable model of factors associated with vaginal birth after cesarean.

Characteristic	Total N = 49	Vaginal Birth After Cesarean (n = 16, 32.7%)	Repeat Cesarean Birth (n = 33, 67.4%)	P-Value

**Sociodemographic**				

Age in years, Median (IQR)	25 [22, 29]	21 [24.5, 29]	27 [23, 29]	0.54^a^
Missing	0 (0.0%)	0 (0.0%)	0 (0.0%)	

Education				0.12^b^
Unable to read & write	7 (14.3%)	1 (6.3%)	6 (18.2%)	
Read & write only	4 (8.2%)	3 (18.7%)	1 (3.0%)	
Primary school	22 (44.9%)	7 (43.7%)	15 (45.5%)	
Secondary school	5 (10.2%)	0 (0.0%)	5 (15.2%)	
Higher education	11 (22.5%)	5 (31.3%)	6 (18.2%)	
Missing	0 (0.0%)	0 (0.0%)	0 (0.0%)	

Religion				0.17^b^
Muslim	8 (16.3%)	1 (6.3%)	7 (21.2%)	
Orthodox Christian	19 (38.8%)	5 (31.2%)	14 (42.4%)	
Catholic Christian	0 (0.0%)	0 (0.0%)	0 (0.0%)	
Protestant	21 (42.9%)	9 (56.2%)	12 (36.4%)	
Jehovah’s Witness	1 (2.0%)	1 (6.3%)	0 (0.0%)	
Missing	0 (0.0%)	0 (0.0%)	0 (0.0%)	

Relationship Status				-
Single	0 (0.0%)	0 (0.0%)	0 (0.0%)	
Not single	49 (100.0%)	16 (100.0%)	33 (100.0%)	
Missing	0 (0.0%)	0 (0.0%)	0 (0.0%)	

Woreda				0.47^c^
Urban	27 (55.1%)	10 (62.5%)	17 (51.5%)	
Rural	22 (44.9%)	6 (37.5%)	16 (48.5%)	
Missing	0 (0.0%)	0 (0.0%)	0 (0.0%)	

Number of Prenatal Visits				0.83^a^
Median (IQR)	4 [4.5]	4 [3.5, 5.5]	4 [4.5]	
Missing	0 (0.0%)	0 (0.0%)	0 (0.0%)	

**Antepartum, Labor, and Delivery**				

Parity				0.29^b^
One	31 (63.3%)	8 (50.0%)	23 (69.7%)	
Two	12 (24.5%)	6 (37.5%)	6 (18.2%)	
Three+	6 (12.2%)	2 (12.5%)	4 (12.1%)	
Missing	0 (0.0%)	0 (0.0%)	0 (0.0%)	

Number of Prior Cesarean Births				1.0^b^
One	44 (89.8%)	15 (98.7%)	29 (87.9%)	
Two	5 (10.2%)	1 (6.3%)	4 (12.1%)	
Missing	0 (0.0%)	0 (0.0%)	0 (0.0%)	

Interpregnancy Interval, Months (IQR)	48 [36, 60]	48 [42, 54]	48 [36,72]	1.0^b^
Missing	0 (0.0%)	0 (0.0%)	0 (0.0%)	

Labor Onset				0.03^b^
Not Applicable	9 (18.4%)	0 (0.0%)	9 (27.8%)	
Spontaneous	39 (79.6%)	16 (100.0%)	23 (69.7%)	
Induced/Augmented	1 (2.0%)	0 (0.0%)	1 (3.0%)	
Missing	0 (0.0%)	0 (0.0%)	0 (0.0%)	

Cervical Exam on Admission				0.009^a^
Median (IQR)	3 [1.5, 3]	3 [2.5, 7]	2 [0.5,3]	
Missing	0 (0.0%)	0 (0.0%)	0 (0.0%)	

Duration of Labor				0.12^b^
Not Applicable	5 (8.9%)	0 (0.0%)	5 (23.8%)	
< 12 hours	26 (46.4%)	18 (51.4%)	8 (38.1%)	
12 – 24 hours	18 (32.1%)	14 (40.0%)	4 (19.1%)	
24+ hours	7 (12.5%)	3 (8.6%)	4 (19.1%)	
Missing	0 (0.0%)	0 (0.0%)	0 (0.0%)	

Antepartum Hemorrhage				0.55^b^
No	53 (94.6%)	34 (97.1%)	19 (90.5%)	
Yes	3 (5.4%)	1 (2.9%)	2 (9.5%)	
Missing	0 (0.0%)	0 (0.0%)	0 (0.0%)	

Antepartum Pre-eclampsia/Eclampsia/Chronic				0.54^b^
Hypertension	46 (93.9%)	16 (100.0%)	30 (90.9%)	
No	3 (6.1%)	0 (0.0%)	3 (9.1%)	
Yes	0 (0.0%)	0 (0.0%)	0 (0.0%)	
Missing				

Gestational Age in Weeks, Median (IQR)	39 [38, 42]	40 [38.5, 41]	39 [38, 42]	0.74^a^
Missing	0 (0.0%)	0 (0.0%)	0 (0.0%)	

Birthweight (grams)				0.33^b^
<2500	1 (2.0%)	1 (6.3%)	0 (0.0%)	
≥ 2500	48 (98.0%)	15 (93.7%)	33 (100.0%)	
Missing	0 (0.0%)	0 (0.0%)	0 (0.0%)	

Baby Sex				0.28^c^
Male	32 (66.6%)	9 (56.3%)	23 (71.9%)	
Female	16 (33.3%)	7 (43.7%)	9 (28.1%)	
Missing	0 (0.0%)	0 (0.0%)	0 (0.0%)	

**Postpartum Complications**				

**Maternal**				

Postpartum Antibiotics				0.02^b^
No	39 (79.6%)	16 (100.0%)	23 (69.7%)	
Yes	10 (20.4%)	0 (0.0%)	10 (30.3%)	
Missing	0 (0.0%)	0 (0.0%)	0 (0.0%)	

**Neonatal**				

Five-Minute Apgar Score Median (IQR)	8 [8, 9]	8 [8, 9]	9 [8, 9]	0.49^a^
Missing	0 (0.0%)	0 (0.0%)	0 (0.0%)	

**Multivariable Model of Characteristics Associated with Vaginal Birth After Cesarean**^d^				

**Characteristic**		**RR**	**CI**	**P-Value**

Onset of Labor		1.7	0.5,5.2	0.37
Centimeters of Cervical Dilation on Admission		1.15	1.1,1.3	0.004

a: Kruskal-Wallis test

b: Fisher’s Exact test

c: Chi-squared test

d: Multivariable Poisson Model with Robust Error Variance of Characteristics Associated with Cesarean Birth
